# Duodenal ulcer accompanied by intractable right lateral chest pain (T6/T7 dermatomal segments)

**DOI:** 10.1186/s40981-016-0038-5

**Published:** 2016-06-04

**Authors:** Yoshimichi Namba, Michiaki Yamakage

**Affiliations:** Sapporo Medical University School of Medicine, Sapporo, Japan

**Keywords:** Duodenal ulcer, Right lateral chest pain

## Abstract

A 48-year-old man who complained of a severe throbbing pain in his right lateral chest was referred to our department. His chest computed tomography (CT) and X-ray, abdominal CT and ultrasonography had revealed no abnormalities. Four days after admission to our ward the patient vomited and he requested upper gastrointestinal (GI) endoscopy: this showed duodenal ulcer. Treatment with omeprazole and sucralfate improved the duodenal ulcer; concurrently, the symptoms of chest pain were relieved.

## Background

Most commonly, the pain of peptic ulcer is referred to the epigastrium [1]. In some patients, the pain of duodenal ulcer is referred to the right upper quadrant, but chest pain is rare [2, 3]. We present a case in which the indicated drug treatment of duodenal ulcer drastically improved a severe right lateral chest pain.

## Case presentation

A 48-year-old man (150 cm, 45 kg) who complained of intractable right lateral chest pain (T6 to T7 dermatomal segments) was hospitalized. Relationship of pain and digestion (oral intake), and time in a day, were variable. The character of pain was “throbbing”. No abnormalities were shown in his chest CT, chest X-ray, abdominal CT, abdominal ultrasonography, rib X-ray. He didn’t have gastroesophageal reflux either. As neither pentazocine nor buprenorphine was able to relieve the pain, the patient was referred from gastroenterology department to our department.

An epidural catheter was inserted at the T6-7 interspace. A continuous infusion of 2 ml of 0.125 % bupivacaine was utilized with bolus dose of 2 ml of 0.25 % bupivacaine for pain control. Effective area of epidural anesthesia was not determined, but epidural block relieved his pain. Results of further investigations using spinal magnetic resonance imaging, intravenous pyelography, renal CT, cardiac enzyme determination were within their normal ranges.

The patient vomited four days after epidural catheterization, and one of the authors was called to see the patient. He had been considering upper GI endoscopy on his way to see the patient, but the patient requested it by himself. The character of pain was changing from “throbbing” to “stabbing or stinging”.

The endoscopy revealed a duodenal ulcer and so the patient was moved to the gastroenterology ward. Omeprazole (proton pump inhibitor) 20 mg daily (orally, once a day) and sucralfate (mucosal protective) 3 g daily (orally, three times a day) were prescribed for 2 weeks. This treatment relieved the patient’s symptoms of chest pain, together with providing the cure for the duodenal ulcer. Fourteen days after commencing the treatment, the patient was discharged and followed as an outpatient for recovery.

## Discussion

The pain of duodenal ulcer is described as “gnawing” or “burning” [3]. The patient described his pain as “throbbing” to “stabbing or stinging”. Most commonly, the pain of duodenal ulcer is referred to the epigastrium [1]. In some patients, the pain is referred to the right upper quadrant. Duodenal ulcer causes pain that radiates to xiphoid process but not higher [2, 3]. Chest pain could be due to peptic ulcer disease if acute ischemic heart disease, pulmonary embolism, aortic dissection and spontaneous pneumothorax were excluded [4]. The patient didn’t have any of the above diseases. Search results through PubMed and UpToDate have not found reports of duodenal ulcer combined with chest pain either.

Since the location of the pain was in the right lateral T6 to T7 dermatomal segments, upper GI endoscopy had not been performed before being referred to our department. Nociceptive impulses from the duodenum are transmitted to the spinal cord through the T5 to T12 [3]. Therefore, pain from the duodenum could radiate to thoracic segments.

## Conclusions

We report a rare case in which the indicated drug treatment of duodenal ulcer drastically relieved a severe pain in the right lateral chest. This case demonstrates that comprehensive examinations are necessary for elucidation of the cause of severe pain.
